# Disease recurrence after colorectal cancer surgery in the modern era: a population-based study

**DOI:** 10.1007/s00384-021-03914-w

**Published:** 2021-04-04

**Authors:** Seyed M. Qaderi, Boris Galjart, Cornelis Verhoef, Gerrit D. Slooter, Miriam Koopman, Robert H. A. Verhoeven, Johannes H. W. de Wilt, Felice N. van Erning

**Affiliations:** 1 Department of Surgical Oncology, Radboud University Medical Center, Nijmegen, The Netherlands; 2 Department of Surgery, Radboud University Medical Center, Geert Grooteplein Zuid 10, 6525 GA Nijmegen, The Netherlands; 3 Department of Surgical Oncology, Erasmus MC Cancer Institute, Rotterdam, The Netherlands; 4 Department of Surgical Oncology, Máxima Medical Center, Eindhoven, The Netherlands; 5 Department of Medical Oncology, University Medical Center Utrecht, Utrecht University, Utrecht, The Netherlands; 6 Department of Research & Development, Netherlands Comprehensive Cancer Organisation, Utrecht, The Netherlands

**Keywords:** Colorectal neoplasm, Recurrence, Locoregional, Distant metastasis, Follow-up studies

## Abstract

**Purpose:**

This population-based study determined the cumulative incidence (CI) of local, regional, and distant recurrences, examined metastatic patterns, and identified risk factors for recurrence after curative treatment for CRC.

**Methods:**

All patients undergoing resection for pathological stage I–III CRC between January 2015 and July 2015 and registered in the Netherlands Cancer Registry were selected (*N* = 5412). Additional patient record review and data collection on recurrences was conducted by trained administrators in 2019. Three-year CI of recurrence was calculated according to sublocation (right-sided: RCC, left-sided: LCC and rectal cancer: RC) and stage. Cox competing risk regression analyses were used to identify risk factors for recurrence.

**Results:**

The 3-year CI of recurrence for stage I, II, and III RCC and LCC was 0.03 vs. 0.03, 0.12 vs. 0.16, and 0.31 vs. 0.24, respectively. The 3-year CI of recurrence for stage I, II, and III RC was 0.08, 0.24, and 0.38. Distant metastases were found in 14, 12, and 16% of patients with RCC, LCC, and RC. Multiple site metastases were found often in patients with RCC, LCC, and RC (42 vs. 32 vs. 28%). Risk factors for recurrence in stage I–II CRC were age 65–74 years, pT4 tumor size, and poor tumor differentiation whereas in stage III CRC, these were ASA III, pT4 tumor size, N2, and poor tumor differentiation.

**Conclusions:**

Recurrence rates in recently treated patients with CRC were lower than reported in the literature and the metastatic pattern and recurrence risks varied between anatomical sublocations.

**Supplementary Information:**

The online version contains supplementary material available at 10.1007/s00384-021-03914-w.

## Introduction

Colorectal cancer (CRC) is the third most common cancer diagnosed worldwide [1, 2]. The numbers of CRC survivors increase due to continuous improvement in all stage survival [3–5].

Despite the increased initial cure rate, 20–30% of patients with stage I–III CRC develop recurrences [3, 6]. This is an important reason for post-treatment surveillance, as it is deemed beneficial to detect and treat disease recurrence at an early stage [3, 7]. In the Netherlands, CRC follow-up occurs according to the national guidelines. [Supplement 1, online only] Up-to-date and specific information regarding the risk of recurrence and prognosis is necessitated for the increasing numbers of CRC survivors at risk for recurrence. Up-to-date information improves patient communication, facilitates optimal shared decision-making with regard to follow-up, and can be used for life planning [8, 9]. Currently, several risk factors for predicting the risk of recurrence are available [8–10]. Besides known tumor-related factors such as pathological stage and histological subtype [11], primary tumor location has emerged as an important prognosticator in recent years [12]. More information about the role of primary tumor location or sidedness, stage, and other potential factors as prognosticators might help to identify patients at high risk for recurrence. In order to improve risk-stratification and information provision in CRC patients, recent population-based evidence on the incidence and location of disease recurrence, and risk factors for recurrence are needed. Studies evaluating the risk of disease recurrence are constrained by the use of data from more than 10 years ago, by using data from relatively small number of patients, or by using data from selective centers and geographical regions [6, 8, 9, 13–15]. Also, treatment of colorectal cancer has changed in the last decade with significant improvements in overall survival [4]. Moreover, national screening programs have been started which led to an increasing number of early colorectal cancers that can often be treated with local endoscopic techniques [16, 17]. Finally, type and location of metastases are usually not reported in previous studies [8, 9, 13]. Therefore, a great need remains for up-to-date, recent, and nationwide data on disease recurrence in patients with CRC.

The aim of this population-based study was therefore to determine the cumulative incidence (CI) and patterns of recurrences in a large and recent cohort of patients with stage I–III CRC. Also, various potential risk factors for recurrence were identified in multivariable analyses.

## Method

### Data collection

Patient sociodemographic and tumor and treatment-related information at time of diagnosis were collected from the Netherlands Cancer Registry (NCR) which covers all newly diagnosed malignancies in the Netherlands. Information is routinely extracted from the medical records by trained administrators of the NCR. Anatomical site of the tumor and metastases is registered according to the International Classification of Disease-Oncology (ICD-O). The UICC TNM (tumor-node-metastasis) classification (7th edition) was used for stage notification of the primary tumor. Comorbidity is registered according to a slightly modified version of the Charlson Comorbidity Index. Additional patient record review and data collection on recurrences was conducted between February and October 2019. Metachronous recurrence was defined as diagnosis of recurrence following resection of the CRC. Information regarding the development of initial (first) recurrences was obtained and encompassed local, regional, and distant recurrences. Local recurrences were defined as recurrences in or near the site of the original primary tumor. Regional recurrences were defined as recurrences in lymph nodes that would classify as regional lymph nodes according to TNM classification. Distant recurrences were defined as recurrences that would be defined as distant metastases according to TNM.

### Study population

This study included all patients in the Netherlands who were diagnosed between January 1st and June 30th, 2015, and that were operated with curative intent for (y) pathological stage I–III primary CRC. Treatment options for tumor’s location in the colon were resection of the primary tumor (both endoscopic and surgical) with or without adjuvant chemotherapy. Treatment options for tumor’s location in the rectum were neoadjuvant therapy consisting of short-term radiotherapy or long course chemoradiation followed by resection of the primary tumor (both endoscopic and surgical) or resection only. Sublocation of the primary tumor was categorized as right-sided colon (RCC): coecum to the splenic flexure (C18.0, C18.2-5), left-sided colon (LCC): splenic flexure to rectum (C18.6-7, incl. rectosigmoid (C19.9)) and rectum (RC, C20.9). Tumors located in the appendix and neuroendocrine tumors were excluded due to different morphological, clinical, and prognostic features than colon and rectal tumors. [18] In case of multiple tumors per patient, only the tumor with the most advanced stage was included. In case pathological stage was unknown or missing, clinical stage was used. Morphology of the tumor was divided into adenocarcinoma (ICD-O codes 8140-1, 8144-5, 8210-1, 8213, 8220-1, 8255, 8261-3), mucinous adenocarcinoma (8470, 8480-1), and signet ring cell carcinoma (8490). Location of distant recurrences was categorized into the liver (C22), lung (C34), peritoneum (C48), and others.

### Statistical analyses

Descriptive statistics, chi-square, and Fisher’s exact test were used to provide an overview of the study population and compare metastatic patterns by sublocation (RCC vs. LCC vs. RC). After stratification by sublocation, the cumulative incidence (CI) functions for recurrence at 3 years were calculated with death as competing event. Because death is an event that may precede recurrence, and therefore might prevent recurrences from occurring and being observed, this is a situation of competing risks. Multivariable Cox competing risk regression models were used to produce cause-specific and independent hazard ratios for recurrence in the presence of dying as a competing risk, for the different patient, tumor, and treatment characteristics. Risk factor for recurrence was identified based on the existing literature and entered into the regression analysis at once. Follow-up assessment began at the date of resection of the primary tumor and ended at the date of diagnosis of recurrence or date of death. Patients without a recurrence or death, or who died after the follow-up period for which information on recurrence was available, were censored at time of last follow-up date for recurrence. Last follow-up date for recurrence differed between patients and was dependent on last patient contact and ascertainment of recurrence status.

*P*-values below 0.05 were considered statistically significant. SAS/STAT® statistical software (SAS system 9.4, SAS Institute, Cary, NC) was used for all analyses.

## Results

### Baseline characteristics

A total of 5412 patients with CRC were included. [Supplement 2, online only] More than two-third (*N* = 3779 patients, 70%) were patients with colon cancer, of which 1807 (34%) had a right-sided tumor and 1972 (36%) a left-sided tumor. Patients with RCC were generally older and had more comorbidities. An overview of patient and tumor characteristics is presented in Table 1***.***

**Table 1:** Sociodemographic, tumor, and treatment characteristics of patients with right-sided and left-sided colon cancer (RCC, LCC) and rectal cancer (RC)

	RCC *N* = 1807	LCC *N* = 1972	RC *N* = 1633	
Gender				
Male Female	872 (48%) 935 (52%)	1196(61%) 776 (39%)	1070 (66%) 563 (34%)	
Age				
<65 years 65–74 years ≥75 years	393 (22%) 731 (40%) 683 (38%)	671 (34%) 829 (42%) 472 (24%)	621 (38%) 641 (39%) 371 (23%)	
Number of comorbidities				
0 1 ≥2 Unknown	783 (43%) 538 (30%) 300 (17%) 186 (10%)	982 (50%) 524 (26%) 233 (12%) 233 (12%)	844 (52%) 411 (25%) 190 (12%) 188 (11%)	
ASA classification				
ASA I ASA II ASA III ASA IV ASA unknown	217 (12%) 937 (52%) 401 (22%) 26 (1%) 226 (13%)	349 (18%) 969 (49%) 277 (14%) 16 (1%) 361 (18%)	319 (20%) 881 (54%) 218 (13%) 6 (<1%) 209 (13%)	
(y) pT stage				
0 1 2 3 4 Unknown	2 (<1%) 187 (11%) 306 (17%) 1049 (58%) 255 (14%) 8 (<1%)	0 (0%) 552 (28%) 341 (17%) 855 (43%) 191 (10%) 33 (2%)	119 (7%) 342 (21%) 506 (31%) 614 (38%) 38 (2%) 14 (1%)	
(y) pN stage				
0 1 2 Unknown	1178 (65%) 401 (22%) 211 (12%) 17 (1%)	,221 (62%) 414 (21%) 197 (10%) 140 (7%)	1154 (71%) 303 (19%) 122 (7%) 54 (3%)	
(y) pTNM stage				
I II III	441 (24%) 754 (42%) 612 (34%)	813 (41%) 548 (28%) 611 (31%)	845 (52%) 363 (22%) 425 (26%)	
Morphology				
Adenocarcinoma Mucinous adenocarcinoma Signet ring cell carcinoma	1541 (85%) 242 (14%) 24 (1%)	1857 (94%) 108 (6%) 7 (<1%)	1549 (95%) 73 (4%) 11 (1%)	
Differentiation grade				
Well/moderate Poor/undifferentiated Unknown	1370 (76%) 221 (12%) 216 (12%)	1745 (88%) 90 (5%) 137 (7%)	1337 (82%) 73 (4%) 223 (14%)	
Microsatellite status				
Stable Instable Unknown	230 (13%) 102 (6%) 1475 (81%)	315 (16%) 21 (1%) 1636 (83%)	154 (9%) 8 (1%) 1471 (90%)	
Treatment				
Neoadjuvant radiotherapy + surgical resection		2 (<1%)	332 (20%)	
Neoadjuvant chemoradiation + surgical resection		4 (<1%)	519 (32%)	
Surgery	1345 (74%)	1436 (73%)	758 (46%) 24 (2%)	
Surgical resection + adjuvant chemotherapy	462 (26%)	530 (27%)		
Type of surgery				
Surgical resection Endoscopic resection	1299 (97%) 46 (3%)	1165 (81%) 271 (19%)	542 (72%) 216 (28%)	
Residual tumor				
No Yes Unknown	1739 (96%) 29 (2%) 39 (2%)	1863 (94%) 39 (2%) 70 (4%)	1519 (93%) 66 (4%) 48 (3%)	

Left-sided included also rectosigmoidal tumors (*N* = 43). (y)pT0N0 was counted as stage I disease and (y)pT0N1-2 as stage III

Comparison of baseline characteristics statistically significant with chi-square tests (*P* < 0.0001)

### Incidence of recurrences

**Fig. 1:**
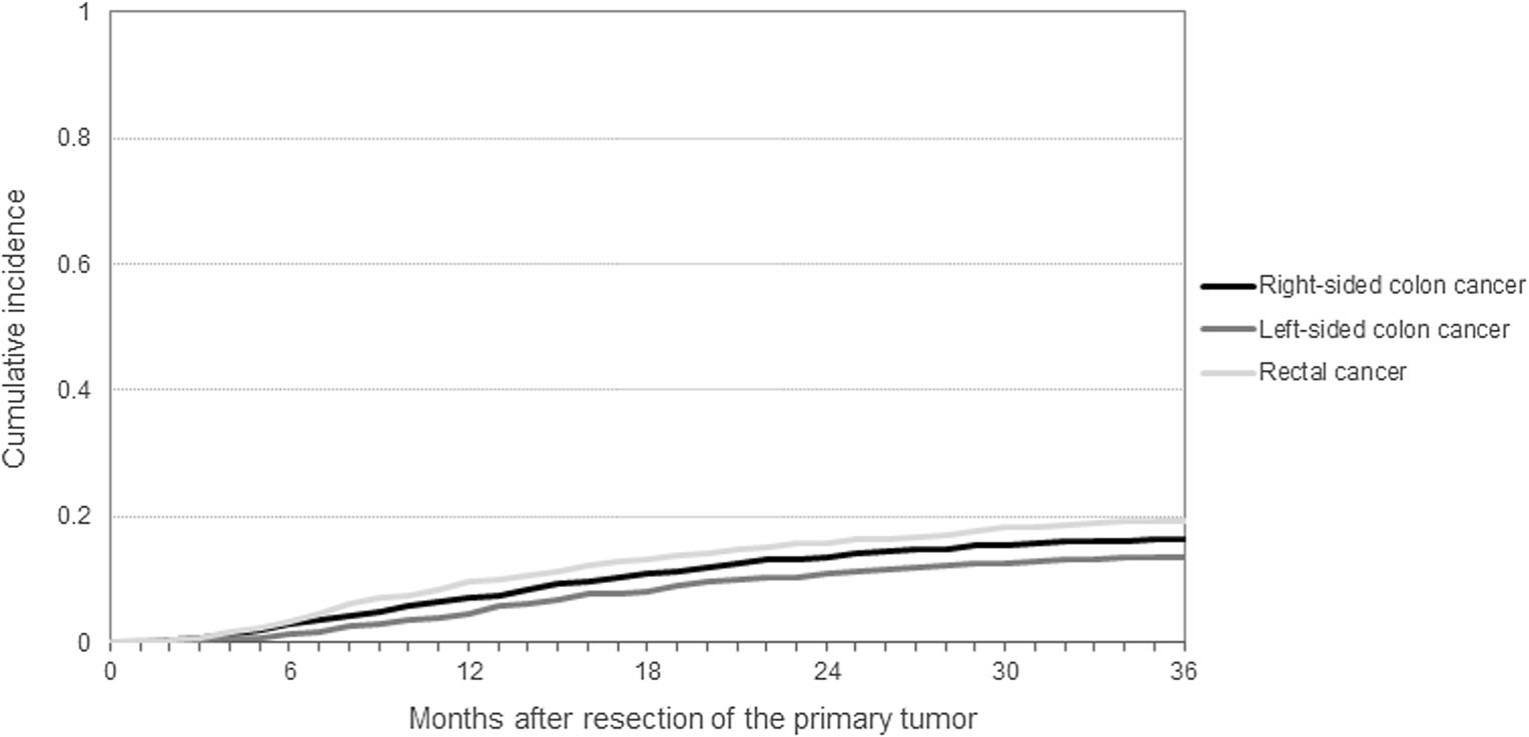
Three-year cumulative incidence of recurrence among patients with right-sided colon and left-sided colon and rectal cancer. Gray’s test: *P* < 0.0001

**Fig. 2:**
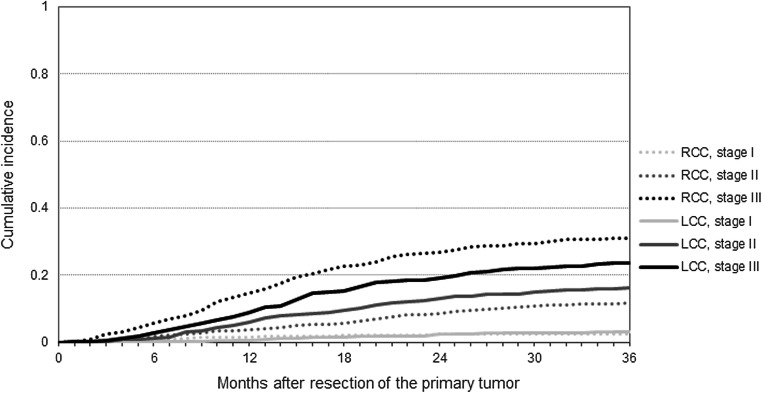
Three-year cumulative incidence of recurrence by primary tumor stage among patients with right-sided colon and left-sided colon cancer. Gray’s test: *P* < 0.0001

**Fig. 3:**
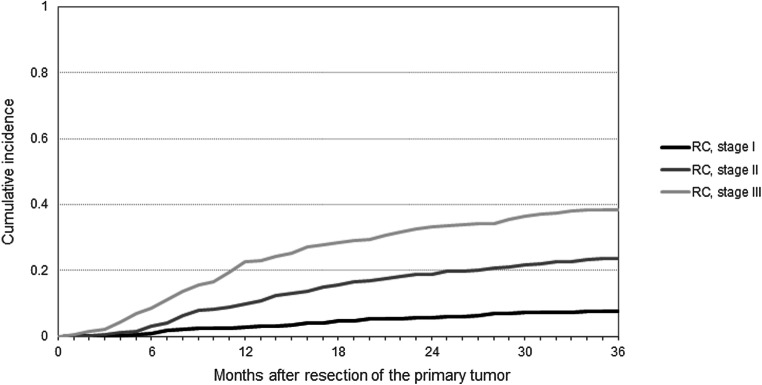
Three-year cumulative incidence of recurrence by primary tumor stage among patients with rectal cancer. Gray’s test: *P* < 0.0001

After a median follow-up of 40 months (IQR 20–46), 877 (16%) recurrences were diagnosed: 287 (16%) among patients with RCC, 262 (13%) among patients with LCC, and 328 (20%) among patients with RC. Median time to recurrence was 14 months (IQR 8–22 months) and 15 months (IQR 10–24 months) for RCC and LCC, and 13 months (IQR 7–23 months) for RC. A total of 34 recurrences were diagnosed within 3 months of follow-up. Differences in the CI of recurrence between patients with RCC, LCC, and RC at 3 years postoperatively were statistically significant (Fig. 1) (Gray’s test: *P* < 0.0001). The 3-year CI of recurrence for patients with stage I, II, and III RCC was 0.03, 0.12, and 0.31, of LCC 0.03, 0.16, and 0.24, and of RC 0.08, 0.24, and 0.38, respectively. (Figs. 2 and 3) Respectively 42, 78, and 93% of the RCC recurrences and 34, 76, and 92% of the LCC recurrences were diagnosed within 1, 2, and 3 years. For the RC recurrences, these percentages were 46, 75, and 90%, respectively. Death without (known) recurrence occurred in 75 (4.9%), 62 (3.6%), and 40 (3.1%) patients with RCC, LCC, and RC, respectively.

Among endoscopically treated patients with a pT1 CRC (*N* = 466, 87% of the total endoscopic group), a total of 19 (4.1%) recurrences were diagnosed after a median follow-up of 40 months (IQR 20–46). There were 2 (0.4%) among patients with RCC, 7 (1.5%) among patients with LCC, and 10 (2.1%) among patients with RC. [Supplement 3, online only] In the remaining endoscopically treated group (*N* = 67) with a pT2Nx or higher CRC, a total of 9 recurrences occurred, 1 (1.5%) among patients with LCC and 8 (11.9%) among patients with RC.

### Risk factors for disease recurrence

In patients with stage I–II RCC and LCC, pT4 tumor size, age 65–74 years, and poor tumor differentiation were associated with a higher risk of recurrence, (Table 2) female gender and was associated with a lower risk of recurrence. ASA III, pT4 tumor size, N2 stage, and poor tumor differentiation were associated with a higher risk of recurrence in patients with stage III RCC or LCC (in which ASA classification was not statistically significant). (Table 3) On the other hand, microsatellite instability and adjuvant chemotherapy in stage III RCC and adjuvant chemotherapy in stage III LCC were associated with a lower risk of recurrence.

**Table 2:** Cumulative incidence of recurrence and hazards for recurrence among patients with right-sided colon cancer

	Stage I–II (*N* = 1195)		Stage III (*N* = 612)	
	Crude 3-year cumulative incidence	Adjusted HR* (95% CI)	Crude 3-year cumulative incidence	Adjusted HR* (95% CI)
Gender				
Male Female	0.10 0.07	1.00 (reference) **0.56 (0.36–0.86)**	0.30 0.32	1.00 (reference) 1.05 (0.78–1.41)
Age				
<65 years 65–74 years ≥75 years	0.06 0.09 0.10	1.00 (reference) **2.00 (1.07–3.72)** 1.75 (0.93–3.28)	0.25 0.32 0.35	1.00 (reference) 1.14 (0.77–1.68) 0.88 (0.55–1.42)
ASA classification				
ASA I ASA II ASA III ASA IV	0.08 0.09 0.10 n.r.	1.00 (reference) 0.82 (0.43–1.58) 0.80 (0.39–1.61) n.r.	0.19 0.29 0.39 n.r.	1.00 (reference) 1.56 (0.94–2.61) **1.85 (1.05–3.26)** n.r.
pT stage				
1 2 3 4	0.03 0.03 0.09 0.31	0.41 (0.16–1.04) **0.32 (0.15–0.69)** 1.00 (reference) **4.84 (2.88–8.12)**	n.r. 0.15 0.27 0.48	n.r. 0.55 (0.27–1.14) 1.00 (reference) **1.90 (1.39–2.59)**
pN stage				
0 1 2	0.09 n.a. n.a.	1.00 (reference) n.a. n.a.	n.a. 0.24 0.45	n.a. 1.00 (reference) **2.03 (1.49–2.76)**
Morphology				
Adenocarcinoma Mucinous adenocarcinoma Signet ring cell carcinoma	0.09 0.03 n.r.	1.00 (reference) 0.51 (0.15–1.69) n.r.	0.30 0.32 0.56	1.00 (reference) 1.04 (0.60–1.80) 1.37 (0.54–3.49)
Differentiation grade				
Well/moderate Poor/undifferentiated	0.08 0.16	1.00 (reference) **2.14 (1.22–3.76)**	0.26 0.43	1.00 (reference) **1.83 (1.26–2.65)**
Microsatellite status				
Stable Instable	0.12 0.04	1.00 (reference) 0.31 (0.07–1.45)	0.36 0.19	1.00 (reference) **0.42 (0.19–0.91)**
Treatment				
Surgery Surgery + adjuvant chemotherapy~	0.08 0.15	Not included	0.37 0.28	1.00 (reference) **0.61 (0.41–0.93)**

ASA classification unknown, T stage unknown, N stage unknown, other morphology, unknown differentiation grade, and unknown microsatellite status were included in the analyses but results not shown

*HR*, hazard ratio; *CI*, confidence interval; *n.a.*, not applicable; *n.r.*, not reported, numbers too small

To prevent problems with multicollinearity between pT, pN, and TNM stage in the multivariable model, TNM stage is not included in the multivariable model

*Competing risk analysis for death as competing event that prevents CRC recurrence from occurring. Hazard ratios are cause-specific hazards for recurrence in the presence of the competing risk of dying

~ In the Netherlands, adjuvant chemotherapy is only recommended for patients with stage III or high-risk stage II colon cancer and not routinely given to rectal cancer patients

**Table 3:** Cumulative incidence of recurrence and hazards for recurrence among patients with left-sided colon cancer

	Stage I–II (*N* = 1361)		Stage III (*N* = 611)	
	Crude 3-year cumulative incidence	Adjusted HR* (95% CI)	Crude 3-year cumulative incidence	Adjusted HR* (95% CI)
Gender				
Male Female	0.08 0.09	1.00 (reference) 1.01 (0.67–1.53)	0.24 0.23	1.00 (reference) 0.87 (0.61–1.25)
Age				
<65 years 65-74 years ≥75 years	0.07 0.09 0.10	1.00 (reference) **1.71 (1.07–2.75)** 1.34 (0.77–2.32)	0.24 0.21 0.28	1.00 (reference) 0.82 (0.55–1.23) 0.65 (0.39–1.08)
ASA classification				
ASA I ASA II ASA III ASA IV	0.11 0.08 0.14 n.r.	1.00 (reference) 0.61 (0.36–1.02) 1.09 (0.60–2.00) n.r.	0.18 0.25 0.23 n.r.	1.00 (reference) 1.51 (0.92–2.49) 1.12 (0.55–2.27) n.r.
pT stage				
1 2 3 4	0.04 0.03 0.14 0.31	**0.33 (0.18–0.59)** **0.25 (0.12–0.50)** 1.00 (reference) **2.71 (1.51–4.89)**	0.09 0.07 0.22 0.46	**0.36 (0.13–0.97)** **0.48 (0.23–0.99)** 1.00 (reference) **2.33 (1.61–3.38)**
pN stage				
0 1 2	0.09 n.a. n.a.	1.00 (reference) n.a. n.a.	n.a. 0.18 0.36	n.a. 1.00 (reference) **2.07 (1.47–2.93)**
Morphology				
Adenocarcinoma Mucinous adenocarcinoma	0.08 0.13	1.00 (reference) 0.82 (0.35–1.94)	0.23 0.31	1.00 (reference) 0.83 (0.34–2.02)
Differentiation grade				
Well/moderate Poor/undifferentiated	0.08 0.20	1.00 (reference) **2.32 (1.07–5.05)**	0.23 0.30	1.00 (reference) 1.30 (0.68–2.51)
Microsatellite status				
Stable Instable	0.13 n.r.	1.00 (reference) n.r.	0.24 0.25	1.00 (reference) 0.70 (0.17–2.98)
Treatment				
Surgery Surgery + adjuvant chemotherapy~	0.08 0.17	Not included	0.35 0.20	1.00 (reference) **0.32 (0.20–0.51)**

ASA classification unknown, T stage unknown, N stage unknown, signet ring cell carcinoma or other morphology, unknown differentiation grade, unknown microsatellite status, neoadjuvant radiotherapy + surgery, and neoadjuvant chemoradiation + surgery were included in the analyses but results not shown

*HR*, hazard ratio; *CI*, confidence interval; *n.a.*, not applicable; *n.r.*, not reported, numbers too small

To prevent problems with multicollinearity between (y)pT, (y)pN, and TNM stage in the multivariable model, TNM stage is not included in the multivariable model

*Competing risk analysis for death as competing event that prevents CRC recurrence from occurring. Hazard ratios are cause-specific hazards for recurrence in the presence of the competing risk of dying

~ In the Netherlands, adjuvant chemotherapy is only recommended for patients with stage III or high-risk stage II colon cancer and not routinely given to rectal cancer patients

All patients with RC who underwent neoadjuvant chemoradiation had an higher risk of recurrence. (Table 4) Those with stage III RC and (y)pT4 tumor size and N2 stage also had an higher risk of recurrence.

**Table 4:** Cumulative incidence of recurrence and hazards for recurrence among patients with rectal cancer

	Stage I–II (*N* = 1208)		Stage III (*N* = 425)	
	Crude 3-year cumulative incidence	Adjusted HR* (95% CI)	Crude 3-year cumulative incidence	Adjusted HR* (95% CI)
Gender				
Male Female	0.14 0.10	1.00 (reference) 0.77 (0.54–1.11)	0.39 0.37	1.00 (reference) 0.89 (0.63–1.24)
Age				
<65 years 65–74 years ≥75 years	0.13 0.10 0.17	1.00 (reference) 0.80 (0.55–1.17) 1.28 (0.83–1.98)	0.41 0.37 0.33	1.00 (reference) 0.94 (0.65–1.36) 0.73 (0.47–1.13)
ASA classification				
ASA I ASA II ASA III ASA IV	0.10 0.13 0.17 n.r.	1.00 (reference) 1.12 (0.70–1.79) 1.73 (0.98–3.05) n.r.	0.41 0.39 0.41 n.r.	1.00 (reference) 0.93 (0.63–1.36) 0.98 (0.56–1.71) n.r.
(y) pT stage				
0 1 2 3 4	0.06 0.05 0.10 0.24 n.r.	**0.20 (0.08–0.47)** **0.27 (0.15–0.46)** **0.51 (0.35–0.74)** 1.00 (reference) n.r.	0.19 0.13 0.21 0.44 0.71	0.33 (0.09–1.12) **0.25 (0.07–0.87)** **0.45 (0.28–0.73)** 1.00 (reference) **2.44 (1.20–4.99)**
(y) pN stage				
0 1 2	0.13 n.r. n.r.	1.00 (reference) n.a. n.a.	n.a. 0.32 0.55	n.a. 1.00 (reference) **1.98 (1.43–2.74)**
Morphology				
Adenocarcinoma Mucinous adenocarcinoma	0.12 0.21	1.00 (reference) 1.40 (0.63–3.13)	0.38 0.38	1.00 (reference) 0.71 (0.33–1.53)
Differentiation grade				
Well/moderate Poor/undifferentiated	0.13 0.19	1.00 (reference) 1.38 (0.60–3.20)	0.36 0.56	1.00 (reference) 1.43 (0.85–2.41)
Treatment				
Neoadjuvant RT + surgery Neoadjuvant CRT + surgery Surgery Surgery + adjuvant chemotherapy~	0.10 0.17 0.11 n.r.	0.78 (0.48–1.27) **1.51 (1.02–2.24)** 1.00 (reference) n.r.	0.34 0.52 0.29 0.15	1.23 (0.81–1.86) **1.76 (1.20–2.60)** 1.00 (reference) n.r.

ASA classification unknown, T stage unknown, N stage unknown, signet ring cell carcinoma or other morphology, unknown differentiation grade, and residual tumor unknown were included in the analyses but results not shown

*RT*, radiotherapy; *CRT*, chemoradiation; *HR*, hazard ratio; *CI*, confidence interval; *n.a.*, not applicable; *n.r.*, not reported, numbers too small

To prevent problems with multicollinearity between pT, pN, and TNM stage in the multivariable model, TNM stage is not included in the multivariable model

*Competing risk analysis for death as competing event that prevents CRC recurrence from occurring. Hazard ratios are cause-specific hazards for recurrence in the presence of the competing risk of dying

~ In the Netherlands, adjuvant chemotherapy is only recommended for patients with stage III or high-risk stage II colon cancer and not routinely given to rectal cancer patients

### Metastatic patterns

CRC metastasized most commonly to other organs. Among patients with a recurrence, respectively, 14, 12, and 16% of patients with RCC, LCC, and RC developed distant metastases. (Table 5) Local recurrences were found more often in patients with RC compared to patients with RCC or LCC (6 vs. 3 vs. 3%, *P* < 0.0001). Among patients with recurrences, the relative proportion of multiple site metastases was higher in patients with RCC, compared to patients with LCC and RC (42 vs. 32 vs. 28%, *P* < 0.0001). In patients with RCC, most distant metastases were found at multiple sites at initial presentation, followed by metastasis in the liver only (27%). In patients with LCC or RC, liver only metastases were most common (39 and 30%). Distant metastases in patients with RC were found most often in the liver (30%), at multiple sites (28%), or in the lungs (28%) at initial presentation. Peritoneal metastases were more common in patients with RCC (33%), compared to LCC and RC (24 and 9%). Localizations of recurrences for endoscopically treated patients can be viewed in Supplement 3 (online only).

**Table 5:** Proportion and localization of recurrences at first detection during a median follow-up of 40 months

	RCC *N* = 1807	LCC *N* = 1972	RC *N* = 1633	*P*-value#
Localization				
Local Local only Regional Regional only Local and/or regional, without distant Distant	52 (3%) 16 (1%) 36 (2%) 8 (<1%) 30 (2%) 257 (14%)	59 (3%) 25 (1%) 23 (1%) 0 31 (2%) 231 (12%)	106 (6%) 54 (3%) 35 (2%) 2 (<1%) 67 (4%) 261 (16%)	<0.0001 <0.0001 0.049 0.003 <0.0001 0.0009
Most common distant localizations*				
Liver Lung Peritoneum Other^	131 (51%) 59 (23%) 86 (33%) 110 (43%)	138 (60%) 56 (24%) 56 (24%) 76 (33%)	114 (44%) 128 (49%) 24 (9%) 72 (28%)	0.002 <0.0001 <0.0001 0.001
Single versus multiple distant localizations*				<0.0001
Liver only Lung only Peritoneum only Other localization only Multiple localizations	68 (27%) 15 (6%) 32 (12%) 34 (13%) 108 (42%)	90 (39%) 17 (7%) 26 (11%) 24 (10%) 74 (32%)	79 (30%) 72 (28%) 11 (4%) 26 (10%) 73 (28%)	

*Proportions calculated for patients with distant recurrence only. *RCC*, right-sided colon cancer, *LCC*, left-sided colon cancer, *RC*, rectal cancer

^Other distant localizations according to ICD-0 topography were small intestine, gallbladder, pancreas, heart/pleura, bones/joints, spleen, skin, soft tissues, cervix, ovary, male genital organs, ureter, bladder, brain, thyroid gland, adrenal gland, ill-defined sites, and lymph nodes

^#^*P*-value indicates significance of chi-square test or Fisher’s exact test as appropriate

## Discussion and conclusions

This comprehensive study presented accurate and recent population-based recurrence data after primary surgical treatment for stage I–III CRC. After a median follow-up of 40 months, 16, 13, and 20% of the patients with RCC, LCC, and RC presented with disease recurrence. The 3-year CI of recurrence for patients with stage I, II, and III RCC and LCC was 0.03 vs. 0.03, 0.12 vs. 0.16, and 0.31 vs. 0.24, respectively. The 3-year CI of recurrence for patients with stage I, II, and III RC was 0.08, 0.24, and 0.38, respectively. Median time to recurrence was approximately 14 months and only differed marginally between the different tumor locations.

Several population-based studies report on the incidence of recurrence in patients with CRC [6, 10, 15, 19–23]. All of these studies only included patients treated before 2012. Since then, several advancements in the treatment of CRC emerged, and postoperative mortality [24] and overall survival [25] have significantly improved. Recurrences rates in the present study for LCC (13%), RCC (16%), and RC (20%) were lower than reported in the largest and most recent population-based study by Holmes et al. [19] Especially in stage I–II disease, CI of recurrence was lower than reported in other studies [26, 27]. In other population-based studies, recurrence rates varied between 12 and 32% for colon cancer [10, 15, 20–22, 28] and between 19 and 31% for rectal cancer [20, 23, 29]. Recurrence rates in the recent randomized FACS and COLOFOL trials were similar to those in the current study [30, 31]. In line with the literature [15, 29], this study showed a decline in the incidence of recurrences in the whole CRC population treated for non-metastatic CRC. Many advances in preoperative, intra-operative, and postoperative treatments could have contributed to the lower recurrence rates. First, better and more accurate disease staging, and patient selection may add to the decline in recurrences [1]. Second, screening programs influenced the total recurrence rate due to shift in stage distribution [32]. In the present study, more than two-thirds of the patients presented with stage I or II while in other studies this was generally lower [8, 13]. Third, an increasing number of early CRCs are sometimes eligible for endoscopic resection [17]. The recurrence rates after endoscopic resection for T1 tumors in the current study were low compared to the literature [16, 33]. However, recurrences rates were high in the group with a pT2Nx rectal cancer patients. This small group of 67 patients was most likely older, more comorbid or ill patients that were found ineligible for a completion TME resection. Fourth, total mesorectal excision (TME) in rectal cancer improved the number of complete circumferential resection margins and as a result lowered the risk of recurrence [34]. Lastly, adjuvant chemotherapy has further targeted microscopic residual disease in colon cancer patients [1, 35]. Adjuvant chemotherapy was associated with lower risk for disease recurrence in both patients with stage III RCC and LCC. The role of adjuvant chemotherapy in stage III colon cancer has been established but its role in rectal cancer and high-risk stage II colorectal cancer is debated [36]. In the Netherlands, adjuvant chemotherapy is only given to a small proportion of stage II colon patients with high-risk characteristics [37]. Since only few of them were treated with adjuvant chemotherapy in the present cohort, we did not include adjuvant chemotherapy in the stage I–II analyses. Neoadjuvant chemoradiation therapy in patients with stage I–II and III RC was associated with a higher risk of recurrence in the present study. This can be explained because neoadjuvant chemotherapy has predominantly been given to patients with locally advanced rectal cancer (LARC). Due to the biological behavior of these advanced tumors, these patients generally have a higher risk to develop metastases compared to more early rectal cancer, despite the neoadjuvant treatment.

The anatomical location of the primary tumor has been demonstrated to be important for survival. Right-sided colon tumors have been associated with an impaired prognosis in both patients with metastatic and non-metastatic disease [12, 38]. An important explanation for this might be the immunopathological and genetic differences between the molecular subtypes of CRC [39]. Population-based studies comparing the incidence of recurrence for sublocations of CRC after curative treatment are, however, rare. Benedix et al. [10] and Manfredi et al. [15] previously observed no difference in RFS between RCC and LCC. Both studies reported data of patients diagnosed between 1976 and 2004 and did not take death into account as a competing risk. Patients with RC were not evaluated in these studies, and their data did not account for the improvements made in diagnosis, surgery, and pre- and postoperative chemotherapy. Van Gestel et al. [14, 40] demonstrated a difference in time to recurrence between patients with rectal and colon cancer, but did not compare RCC and LCC. Again, competing risks were not taken into account in their analyses. This is especially relevant for the comparison between primary tumor sublocations, as baseline characteristics varied across patients’ groups. In addition, the relative proportion of patients dying without experiencing disease recurrence was twice as high in the RCC subgroup (5.9%), which could relate to the older age and higher ASA score in these patients. Also, some studies suggest that immune response is worse due to senescence [41] and that older age is associated with decreased lymph node yield. [42] This might also have an impact on recurrences during follow-up. Another factor is the ongoing scientific debate regarding lifestyle, comorbidities, and recurrences wherein some studies suggest that comorbidity is also associated risk of recurrence and poorer survival. [43, 44] Moreover, patients with higher ASA scores will less likely be found eligible for adjuvant chemotherapy. Therefore, analyzing disease recurrence in light of these abovementioned competing risks is important. The present study is the first population-based study to compare only CI of recurrence between all sublocations of CRC, including these competing risks.

With regard to the patterns of recurrence, limited data is available. However, primary tumor location seems highly associated with location of recurrences. We demonstrated that distant metastases in patients with RCC occurred more often at multiple sites compared to LCC and RC (42 vs. 32 vs. 28%). When evaluating the sites affected, peritoneal metastases were more often seen in patients with RCC. A large study evaluating recurrence patterns in patients with stage I–III CRC was published by Augestad et al. [22]. The proportion of isolated liver recurrences was higher in LCC (35% of all recurrences), while isolated lung metastases were more often diagnosed in patients with RC (20%). Another study showed notable differences between sublocations in patients with stage I–IV CRC [20].

The findings of this and other studies may have implications in clinical practice. First, these data can be used as indicators of CRC prognosis in the Netherlands and other (Western) countries with similar healthcare systems. The DCRA has audited CRC care in the Netherlands over the last decade and significant improvements in screening [45] and care of patients with CRC [46] were noted, especially in the elderly and those with rectal cancer. Unfortunately, the DCRA does not report on long-term follow-up and as such cannot be used for auditing. Merging the databases of DCRA and NCR could potentially be used for future auditing and feedback of long-term outcome per hospital. Second, results of this study can be used to help inform patients about their risk of developing recurrences. Although based on this study we cannot tailor follow-up frequency and length, the data may be used to provide up-to-date stage- and tumor site–specific information regarding recurrence risk. Together with the patients’ health status and wishes, accurate shared decision-making can be facilitated. Patients with stage I colon cancer in our cohort had a CI of recurrence of 0.03 at 3 years follow-up. Considering the excellent 5-year conditional relative survival of patients with stage I colon cancer [5], this low-risk patient group could be eligible for less intensive, personalized follow-up [47, 48]. Future follow-up will rely more on testing for (bio) markers rather than performing periodic imaging, there may be greater scope for incorporating this care into alternative follow-up settings (i.e., home, primary care) [48]. Personalization of follow-up care by patient factors (i.e., age, comorbidity, mobility) and disease stage could be considered as well after shared decision-making with the patient.

Although this study evaluated disease recurrence in a large population-based cohort of patients, diagnosed and treated according to most recent standards, the study has some limitations. First, the median follow-up time (40 months) in this study was relatively short. Development of disease recurrence after this follow-up time might have been missed. However, it has been shown previously that the vast majority of recurrences occurs within 3 years follow-up [6]. Therefore, it is unlikely that our findings would notably change with longer follow-up. Second, we reported information regarding initial recurrences only while recurrences in other locations during further follow-up might have significant clinical implications.

In conclusion, this comprehensive population-based study provided recent and accurate recurrence data in patients with stage I–III CRC. The 3-year CI of recurrence was lower than reported in the literature and the metastatic pattern and risk of recurrence differed between the anatomical sublocations of CRC. These findings help to assess prognosis and can be used for individualized patient information during follow-up.

## Supplementary Information

ESM 1 (DOCX 12 kb)

ESM 2 (DOCX 45 kb)

ESM 3 (DOCX 30 kb)

## Data Availability

Data are available on reasonable request.
