# Supplementary material for: Disease recurrence after colorectal cancer surgery in the modern era: a population-based study
Source: Int J Colorectal Dis. 2021 Apr 4;36(11):2399–410. doi: 10.1007/s00384-021-03914-w (PMC8505312; doi:10.1007/s00384-021-03914-w)
Supplement: Supplementary file 1 — (DOCX 12 kb) [file 384_2021_3914_MOESM1_ESM.docx]

**Supplement 1. Dutch CRC follow-up guidelines.**

Last update in 2014, the Dutch colorectal cancer guidelines recommends half-yearly outpatient clinic visit for 2-3 years postoperative and yearly hereafter up to 5 years. CEA measuring is recommended 3-6 monthly up to 3 years and half-yearly hereafter. Furthermore, abdominal ultrasound, CT-abdomen and chest X-ray (rectal cancer only) are recommended every half-year up to three years and yearly hereafter. The Dutch Colonoscopy Surveillance guideline recommends colonoscopy at 1-, 3- and 5-years post-operative. In case of incomplete pre-operative colonoscopy, this should be done within 3 months postoperatively.

Weblink reference of the complete guidelines: <https://www.oncoline.nl/colorectaalcarcinoom>
