# Supplementary material for: Disease recurrence after colorectal cancer surgery in the modern era: a population-based study
Source: Int J Colorectal Dis. 2021 Apr 4;36(11):2399–410. doi: 10.1007/s00384-021-03914-w (PMC8505312; doi:10.1007/s00384-021-03914-w)
Supplement: Supplementary file 2 — (DOCX 45 kb) [file 384_2021_3914_MOESM2_ESM.docx]

**Supplement 2** Flowchart

All primary CRC

diagnosed January 1^st^ – June 30^th^ 2015

in the Netherlands

n=7,941

Patients with (y)pathological stage I-III CRC

diagnosed January 1^st^ – June 30^th^ 2015

and operated with curative intent

in the Netherlands

n=5,412

Multiple tumors per patient excluded (except most advanced)

n=61

Not operated with curative intent excluded

n=437

Topography appendix or colon other/NOS excluded

n=56

Synchronous metastasized disease (M1) excluded

n=1,526

Tumors without data on recurrences excluded

n=325

Neuroendocrine tumors and morphology other/NOS excluded

n=124
