# Supplementary material for: Disease recurrence after colorectal cancer surgery in the modern era: a population-based study
Source: Int J Colorectal Dis. 2021 Apr 4;36(11):2399–410. doi: 10.1007/s00384-021-03914-w (PMC8505312; doi:10.1007/s00384-021-03914-w)
Supplement: Supplementary file 3 — (DOCX 30 kb) [file 384_2021_3914_MOESM3_ESM.docx]

**Supplement 3 Recurrences among patients with endoscopic resection**

|  | **Total endoscopic resection group**  **N=533** | | | | **T_1_N_0-X_ endoscopic resection group**  **N=466** | | | |
| --- | --- | --- | --- | --- | --- | --- | --- | --- |
|  | **RCC**  N=46 | **LCC** N=271 | **RC** N=216 | **P-value^#^** | **RCC**  N=41 | **LCC** N=240 | **RC** N=185 | **P-value^#^** |
| **Localization**  Local  Local only  Regional  Regional only  Local and/or regional, without distant  Distant | 2 (4%)  2 (4%)  0 (0%)  0 (0%)  2 (4%)  0 (0%) | 8 (3%)  4 (1%)  4 (1%)  0 (0%)  5 (2%)  3 (1%) | 13 (6%)  6 (3%)  8 (4%)  1 (<1%)  11 (5%)  7 (3%) | 0.254  0.243  0.212  0.492  0.133  0.205 | 2 (5%)  2 (5%)  0 (0%)  0 (0%)  2 (5%)  0 (0%) | 7 (3%)  3 (1%)  4 (2%)  0 (0%)  4 (2%)  3 (1%) | 7 (4%)  3 (2%)  5 (3%)  0 (0%)  6 (3%)  4 (2%) | 0.771  0.274  0.587  1.000  0.244  0.726 |

466 (87.4%) of 533 patients treated with endoscopic resection only had a T_1_N_0-X_

*^#^P-value indicates significance of Chi-square test or Fisher’s Exact test as appropriate.*
